# Alterations of Sub-cortical Gray Matter Volume and Their Associations With Disease Duration in Patients With Restless Legs Syndrome

**DOI:** 10.3389/fneur.2018.01098

**Published:** 2018-12-17

**Authors:** Tian Li, Chunyan Liu, Hanqing Lyu, Zhexue Xu, Qingmao Hu, Bibo Xu, Yuping Wang, Jinping Xu

**Affiliations:** ^1^Department of Psychology, Faculty of Education, Hubei University, Wuhan, China; ^2^Institute of Biomedical and Health Engineering, Shenzhen Institutes of Advanced Technology, Chinese Academy of Sciences, Shenzhen, China; ^3^Department of Neurology, Xuan Wu Hospital, Capital Medical University, Beijing, China; ^4^Beijing Key Laboratory of Neuromodulation, Beijing, China; ^5^Radiology Department, Shenzhen Traditional Chinese Medicine Hospital, Shenzhen, China

**Keywords:** restless legs syndrome, sub-cortical alteration, resting-state functional connectivity, surface-based morphometry, voxel-based morphometry

## Abstract

**Object:** The purpose of this study was to uncover the pathology of restless legs syndrome (RLS) by exploring brain structural alterations and their corresponding functional abnormality.

**Method:** Surface-based morphometry (SBM) and voxel-based morphometry (VBM) were performed to explore the alterations in cortical and sub-cortical gray matter volume (GMV) in a cohort of 20 RLS and 18 normal controls (NC). Furthermore, resting-state functional connectivity (RSFC) was also performed to identify the functional alterations in patients with RLS.

**Results:** We found significant alterations of sub-cortical GMV, especially the bilateral putamen (PUT), rather than alterations of cortical GMV in patients with RLS compared to NC using both SBM and VBM. Further sub-regional analysis revealed that GMV alterations of PUT was mostly located in the left dorsal caudal PUT in patients with RLS. In addition, altered RSFC patterns of PUT were identified in patients with RLS compared to NC. Moreover, correlation analyses showed that the GMV of the left caudate and the left ventral rostral PUT were positively correlated with disease duration in patients with RLS.

**Conclusions:** The alterations of subcortical GMV might imply that the primarily affected areas are located in sub-cortical areas especially in the sub-region of PUT by the pathologic process of RLS, which might be used as potential biomarkers for the early diagnosis of RLS.

## Introduction

Restless legs syndrome (RLS) is a common sensorimotor disorder, which is characterized by a nearly irresistible urge to move legs; the symptoms fluctuate in severity with the diurnal cycle, worsening during the night-time hours (1, 2). Since symptoms usually worsen during the night, which caused sleep problem, it was reported that widespread encephalic regions including areas related to motion, sensation, and arousal (3) were activated in patients with RLS. Although a series of MRI studies have reported structural and functional brain abnormality in patients with RLS using various neuroimaging techniques (4–9), the underlying pathological mechanism remains poorly understood.

Functionally, fMRI studies demonstrated that areas including the somatosensory and sensorimotor related cortices, the cerebellum and the thalamus are activated in patients with RLS (6, 8, 10, 11). Structurally, voxel-based morphometry (VBM) study demonstrated gray matter (GM) changes in various brain areas in patients with RLS. Previously, a VBM study identified increased gray matter volume (GMV) in bilateral thalamic pulvinar nuclei in patients with RLS (12). Later, Unrath et al. (13) demonstrated decreased GMV in the bilateral primary somatosensory cortex and the left-sided primary motor areas in patients with RLS. Hornyak et al. (14) found increased GM density in the ventral hippocampus and middle orbitofrontal gyrus in patients with RLS. Recently, a study found decreased regional GMV in the left hippocampal gyrus, bilateral parietal lobes, medial frontal areas and cerebellum in patients with RLS (15). However, the results of these studies were inconsistent, which might be explained by methodological differences, like the use of the different threshold of probability, various software, as well as differences in clinical parameters including medication status, severity or disease duration (4). Furthermore, few studies have detected changes in a sub-regional level. As one region of the brain usually involves various functions, the sub-regional changes might explain the inconsistence of structural and functional abnormality in patients with RLS. This might also provide insights of which sub-region is affected primarily by the pathologic process and which is involved secondarily in patients with RLS.

Using surface geometry to do inter-subject comparisons of cortical brain areas, surface-based morphometry (SBM) analyzes brain volumes as structures as a whole, which provides a novel methodological outlet to explore the pathophysiological mechanism in patients with RLS (16, 17). Considering the inconsistences might result from the methodological differences, we combined SBM and VBM as well as resting-state functional connectivity (RSFC) to explore the structural and functional alterations in patients with RLS. In addition, in order to detect GMV changes in a sub-regional level, pre-defined atlases were used.

## Materials and Methods

### Participants

Twenty patients fulfilling the International Restless Legs Syndrome Study Group (IRLSSG) diagnostic criteria for idiopathic RLS were recruited in the study (5 male, mean age 56.60 ± 9.86 years) (1). None of them received medical treatments. The disease duration ranges from 1 to 40 years (16.05 ± 12.72). Exclusion criteria were iron deficiency anemia, pregnancy, chronic renal disease, diabetes, peripheral neuropathy, or radiculopathy. Clinical measurements included Restless Legs Syndrome Rating Scale (RS_RLS) and Pittsburgh Sleep Quality Index (PSQI). Eighteen normal controls (NC) (5 male, mean age 57.28 ± 4.63 years) were also involved in the present study. All subjects were given written informed consent, and the study was approved by the Institutional Review Board of the Xuan Wu hospital. The demographic and clinical data were shown in Table 1.

**Table 1:** Demographics and clinical data.

	**RLS**	**NC**	***p-*value**	**ES**
Number	20	18		
Gender (male:female)	5:15	5:13	1.00^a^	0.032^c^
Age (mean ± SD)	56.60 ± 9.86	57.28 ± 4.63	0.79^b^	0.088^d^
Durations (mean ± SD)	16.05 ± 12.72	–	–	
RS_RLS (mean ± SD)	23.25 ± 6.95	–	–	
PSQI (mean ± SD)	11.90 ± 4.02	–	–	

a *χ-test*.

b *Two-sample t-test*.

c *Cramer's V*.

d *Cohen's d*.

*ES, effect size; RLS, Restless Legs Syndrome; NC, Normal Controls; RS_RLS, Rating Scale for RLS; and PSQI, Pittsburgh Sleep Quality Index*.

### MRI Acquisition

MRI scanning was conducted on a 3T scanner (MAGNETOM, Skyra, Siemens, Erlangen, Germany) with a 32-channel head-coil. Anatomic images were acquired in sagittal orientation with three-dimensional inversion recovery prepared fast spoiled gradient recalled sequence (repetition time/echo time ratio = 1,900/2.2 ms, inversion time = 900 ms, flip angle = 9 degrees, matrix size = 256 × 256, slice thickness = 1 mm, voxel size = 1 × 1 × 1 mm^3^, and sections = 176). The resting-state functional images were acquired using a standard echo planar imaging (EPI) sequence (repetition time/echo time ratio = 2,000/40 ms, 239 volumes, flip angle = 90 degrees, 28 slices, thickness/gap = 4.0/1.0 mm, voxel size = 3.75 × 3.75 × 4.00 mm^3^, and matrix size = 64 × 64).

### SBM Analysis

Each T1 scan was processed using the FreeSurfer pipeline, which is a semi-automated approach described in detail in prior publications (18, 19). Briefly, the image processing included skull stripping, automated Talairach transformations, segmentation of the sub-cortical white matter (WM) and deep GM structures, intensity normalization, tessellation of the boundary between GM and WM, automated topology correction, and surface deformation along intensity gradients for optimal placement of the borders between GM, WM, and cerebrospinal fluid. For quality control, the pial and white matter surfaces were then visually inspected for errors, and edited when necessary according to the FreeSurfer editing manual (https://surfer.nmr.mgh.harvard.edu/fswiki/FreeviewGuide/FreeviewWorkingWithData/FreeviewEditingaRecon). As a result, no subjects with serious topological defects in the cortical surfaces were excluded.

The cortical GMV was analyzed using a surface-based group analysis of FreeSurfer's Qdec (version 1.5). First, the spatial cortical GMV was smoothed with a circularly symmetric Gaussian kernel of 10 mm full width half maximum to provide normal distribution of the results. Then, we employed a general linear model (GLM) analysis with age and gender as the nuisance factors in the design matrix to directly compare the GMV of the two groups. Finally, the GLM result was corrected for multiple comparisons utilizing a pre-cached cluster-wise Monte Carlo simulation (20) implemented in Qdec (mc-z, threshold: *p* < 0.001, sign: absolute).

The subcortical GMV including the bilateral thalamus, hippocampus, amygdala, putamen, globus pallidus, and caudate were calculated using the automated procedure for volumetric measurements of brain structures implemented in the FreeSurfer (21). Comparisons in the volumes of these regions were performed using the GLM analysis with age and gender as covariates using IBM SPSS 19.0 (IBM, Armonk, NY).

### VBM Analysis

VBM analysis was also performed to determine brain structural differences between RLS and NC. The structural MRI images were preprocessed using DPABI toolbox (http://rfmri.org/dpabi). Each structural image was segmented into gray matter, white matter and cerebrospinal fluid using a fully automated algorithm within DPABI and subsequently transformed to Montreal Neurological Institute (MNI) space using DARTEL-normalization. Next, the normalized gray matter images were smoothed (FWHM = 6 mm) for statistical analyses (22). The modulated images were used to calculate the GMV and were corrected for total intracranial volume. Finally, two sample *t*-tests were performed on these normalized GMV maps to determine brain regions with significant differences. False discovery error (FDR) correction was used for multi-comparison correction (*p* < 0.05, FDR corrected, cluster size ≥100 voxels).

### Defining Sub-regions of PUT

The three sub-regions of PUT in each hemisphere were defined as 6 mm spherical seeds. The centers of the seeds were: dorsal caudal PUT (dcPUT, ± 28, 1, 3), dorsal rostral PUT (drPUT, ± 25, 8, 6), and ventral rostral PUT (vrPUT, ± 20, 12, – 3) (23).

### Sub-regional GMV Analysis

The modulated images were used to calculate sub-regional GMV of the PUT. Two sample *t*-tests were performed to determine the sub-regional differences, and the significance level was set at *p* < 0.05.

### Resting-State fMRI (rs-fMRI) Data Preprocessing

The preprocessing of the rs-fMRI data was carried out using DPABI toolbox. The first 10 volumes were discarded to allow for magnetization equilibrium. The slice timing for the remaining images was corrected, and images were realigned to the first volume to account for head motion. All participants who showed a maximum displacement of < 3 mm and an angular motion of < 3° were included in the subsequent analyses. All fMRI images were normalized to the MNI template and resampled to 3 × 3 × 3 mm^3^. Subsequently, the functional images were smoothed using a Gaussian kernel of 6 mm FWHM. Finally, the functional images were filtered with a temporal band-path of 0.01~0.1 Hz and six motion parameters, WM and cerebrospinal fluid signals were regressed out. Because previous studies have shown that global mean signal regression can lead to spurious resting-state functional correlations and false inferences, particularly on the group level inference (24, 25), the global mean signal was regressed during the preprocessing in our current study.

### RSFC Analysis

To identify whether functional connectivity was changed in patients with RLS, whole brain RSFC was computed using clusters with significant GMV changes in patients with RLS as seed regions. First, we resampled the seed regions to 3 mm cubic voxels in MNI space. Then, the Pearson correlation coefficients between the mean time series for each seed region and the mean time series for each voxel of the whole brain were calculated for each subject, and then converted to *z-*values using Fisher's *z* transformation to improve normality. Finally, each individual's *z*-values were entered into a two-sample *t*-test on the group level (age and gender as covariant) to determine the regions that showed significantly different connectivity with the seed region. As the false discovery rate (FDR) method is too strict for whole brain functional analysis, the significance was determined using Gaussian random field (GRF) correction voxel level *p* < 0.001, cluster level *p* < 0.05 (26).

### Correlation Analysis

We performed correlation analyses between mean GMV and the clinical scores (RS_RLS, PSQI scores, and disease duration) to further explore whether neuroimaging indices were related to changes on the symptom-level in the patients with RLS. The significance level was set at *p* < 0.05.

## Results

### Demographic Data

There was no significant difference in age and gender between RLS and NC.

### SBM Results

**Figure 1:**
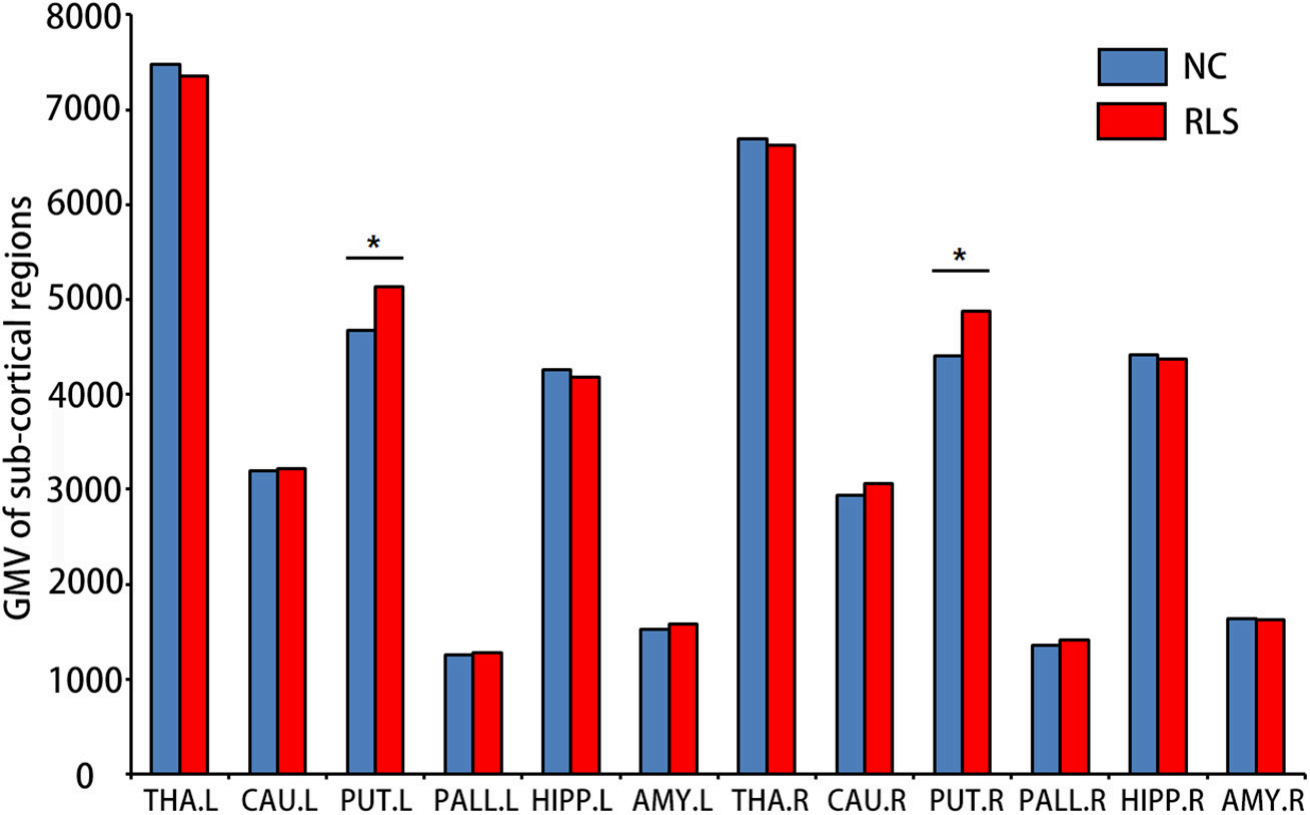
Sub-cortical regions which showed altered gray matter volumes (GMV) using surface-based morphometry (SBM) in patients with restless legs syndrome (RLS) compared to normal controls (NC). Multi-comparison was used to identify the altered GMV (Bonferroni correction, *p* < 0.05). L, left; R, right; THA, thalamus; CAU, caudate; PUT, putamen; PALL, globus pallidus; HIPP, hippocampus; and AMY, amygdala. ^*^means the significant difference between RLS and NC.

Although no significant difference in cortical GMV was found, increased sub-cortical GMV of the left [*F*_(3, 34)_ = 4.174, *p* = 0.049, $\eta_{\text{p}}^{2}$ = 0.109] and the right PUT [*F*_(3, 34)_ = 6.768, *p* = 0.014, $\eta_{\text{p}}^{2}$ = 0.166] were identified in patients with RLS compared to NC (Figure 1).

### VBM Results

**Figure 2:**
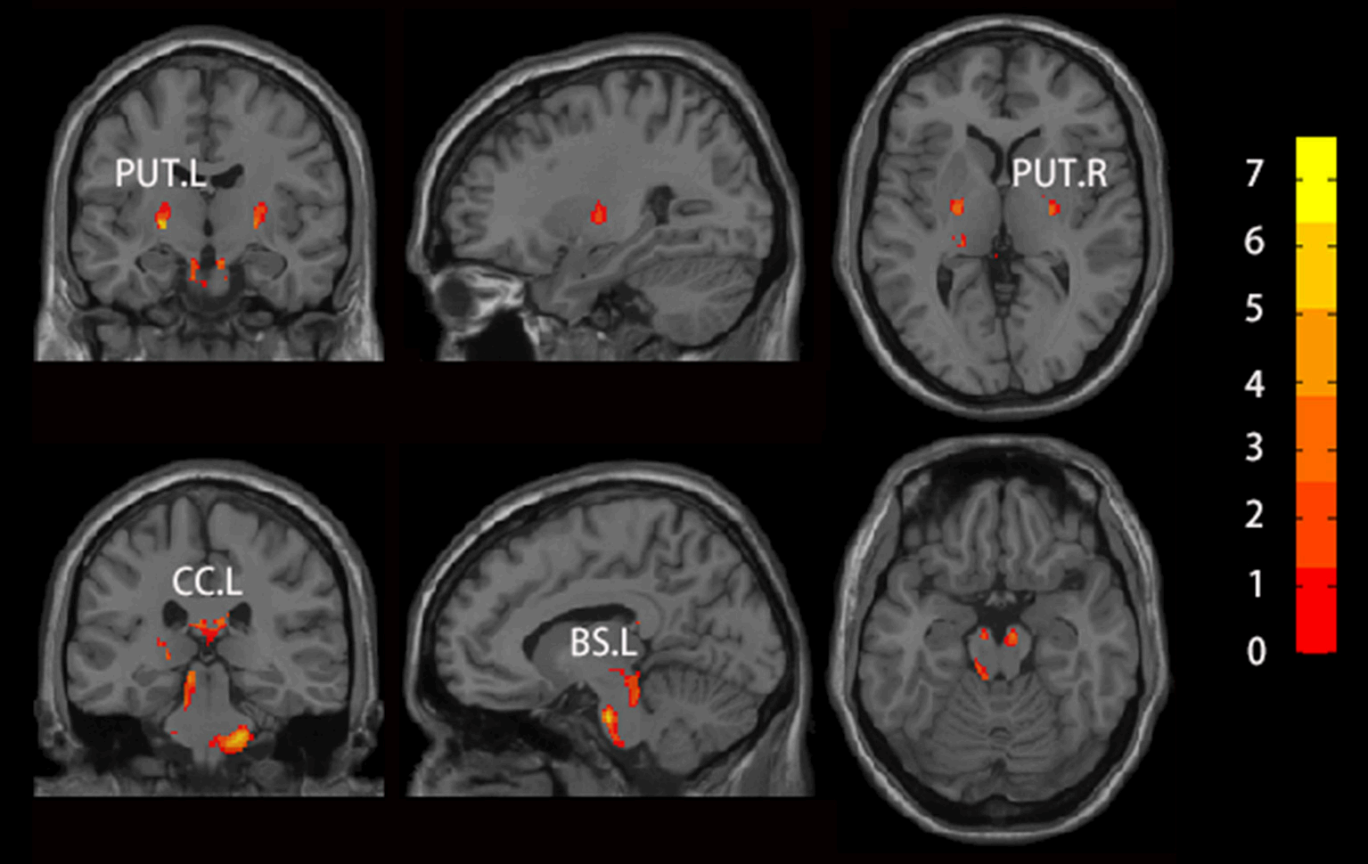
Brain regions which showed altered GMV using voxel-based morphology (VBM) in patients with RLS compared to NC. Two sample t-test was used to identify the changed GMV, and false discovery error (FDR, *p* < 0.05) was used for multi-comparison. Abbreviations were listed in Table 2.

We found increased GMV of the PUT.L and bilateral brainstem in patients with RLS compared to NC (Figure 2, Table 2).

**Table 2:** Brain regions showing significantly altered GMV in patients with RLS using VBM.

**Brain regions**	**Cluster size**	**Peak point coordinates**	**Peak intensity**	**Cohen's f^**2**^**
BS.L	1,449	(−11, −15, −30)	7.175	1.514
BS.L	331	(−9, −32, −9)	6.927	1.411
PUT.L	226	(−24, −11, 0)	7.624	1.709
PUT.R	144	(24, −12, 0)	6.227	1.140
CC.L	389	(−3, −22, 21)	6.213	1.135

*The significance was determined using False discovery error (FDR) correction (p < 0.05, cluster size ≥100 voxels). L, left; R, right; BS, brainstem; PUT, putamen; and CC, corpus callosum*.

### Sub-regional GMV of PUT

**Figure 3:**
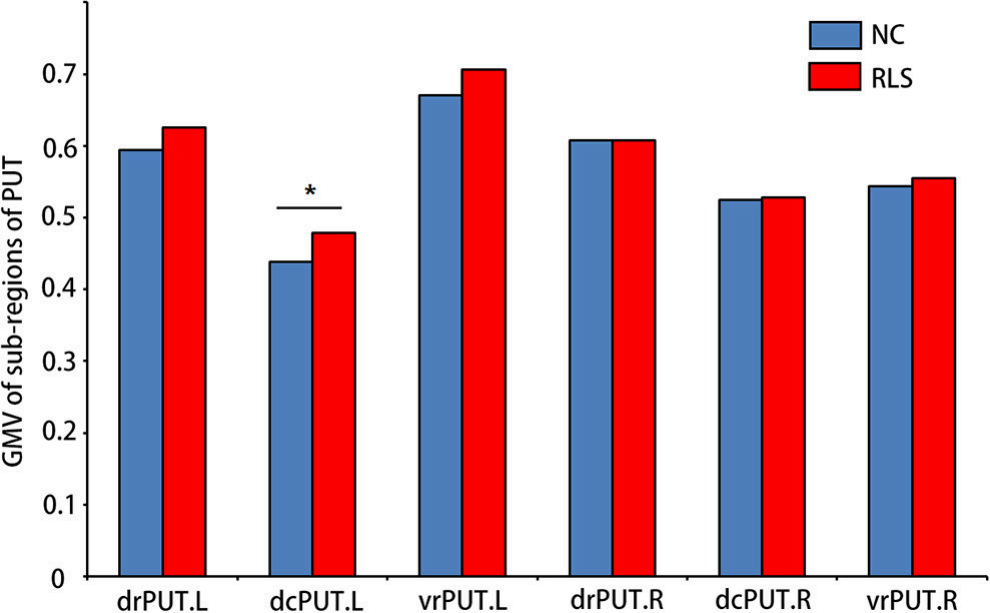
Sub-regions of PUT which showed altered GMV in patients with RLS compared to NC. Two-sample *t*-test was used to identify the changed GMV. dcPUT, dorsal caudal putamen; drPUT, dorsal rostral putamen; and vrPUT, ventral rostral putamen. ^*^means the significant difference between RLS and NC.

Increased GMV of the dcPUT.L (*t* = −2.305, *p* = 0.027, Cohen's d = 0.744) was found in patients with RLS compared to NC (Figure 3).

### RSFC Results

**Figure 4:**
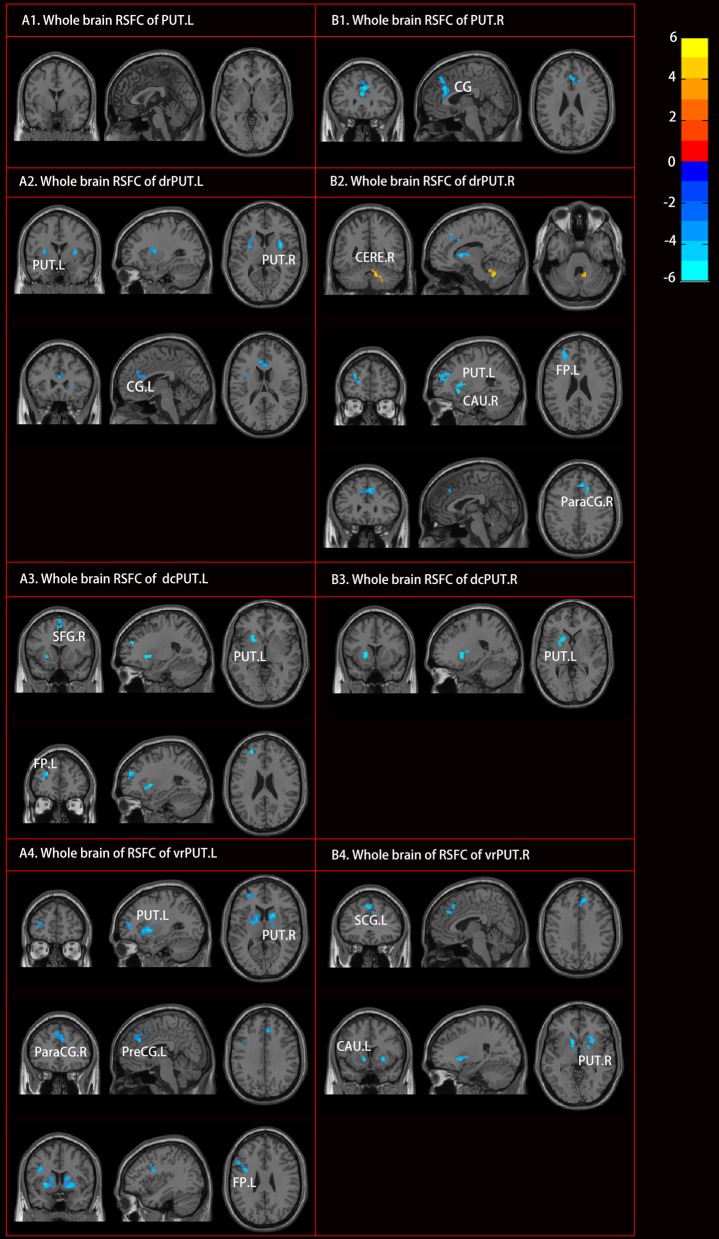
Altered resting-state functional connectivity (RSFC) of PUT in patients with RLS compared to NC. Two sample *t*-tests and Gaussian random field (GRF) correction (voxel level *p* < 0.001, cluster level *p* < 0.05) were used. Abbreviations were listed in Table 3.

Although no RSFC change of the PUT.L was found, altered RSFC of the PUT.R as well as the bilateral sub-divisions, were identified [mostly with the bilateral anterior cingulate gyrus, the left frontal pole, and bilateral caudate (CAU)] in the patients with RLS (Figure 4, Table 3).

**Table 3:** Brain regions showing significantly altered RSFC of PUT in RLS.

**Seed regions**	**Cluster size**	**Peak MNI coordinates**	**Peak intensity**	**Brain regions**	**Cohen's f^**2**^**
PUT.R	184	(0, 30, 18)	−6.021	CG	1.066
drPUT.L	71	(−30, 0, 9)	−5.300	PUT.L	0.826
	83	(33, 3, 9)	−6.939	PUT.R	1.416
	43	(−9, 30, 18)	−5.267	CG.L	0.816
dcPUT.L	37	(−24, 18, −3)	−5.509	PUT.L	0.893
	38	(−27, 51, 24)	−4.942	FP.L	0.718
	52	(3, 12, 69)	−4.957	SFG.R	0.723
vrPUT.L	219	(24, 15, −6)	−6.443	PUT.R	1.221
	196	(−27, 6, −3)	−6.540	PUT.L	1.258
	32	(−30, 48, 9)	−4.618	FP.L	0.627
	56	(−36, 3, 27)	−5.606	PreCG.L	0.924
	122	(6, 30, 33)	−4.962	ParaCG.R	0.724
drPUT.R	54	(12, −51, −33)	6.040	CERE.R	1.073
	104	(−18, 12, 3)	−5.768	PUT.L	0.979
	60	(12, 12, 3)	−5.378	CAU.R	0.851
	73	(−30, 48, 24)	−5.565	FP.L	0.911
	61	(6, 30, 39)	−5.085	ParaCG.R	0.761
dcPUT.R	83	(−21, 12, 3)	−5.288	PUT.L	0.822
vrPUT.R	44	(−12, 12, −3)	−5.444	CAU.L	0.871
	60	(27, 12, −3)	−5.040	PUT.R	0.747
	97	(−3, 33, 45)	−5.987	SCG.L	1.054

*The significance was determined using Gaussian random field (GRF) correction, voxel level p < 0.001, cluster level p < 0.05. MNI, Montreal Neurological Institute; CG, cingulate gyrus; FP, frontal pole; SFG, superior frontal gyrus; CAU, caudate; PreCG, precentral gyrus; ParaCG, paracingulate gyrus; and CERE, cerebellum*.

### Correlation Results

**Figure 5:**
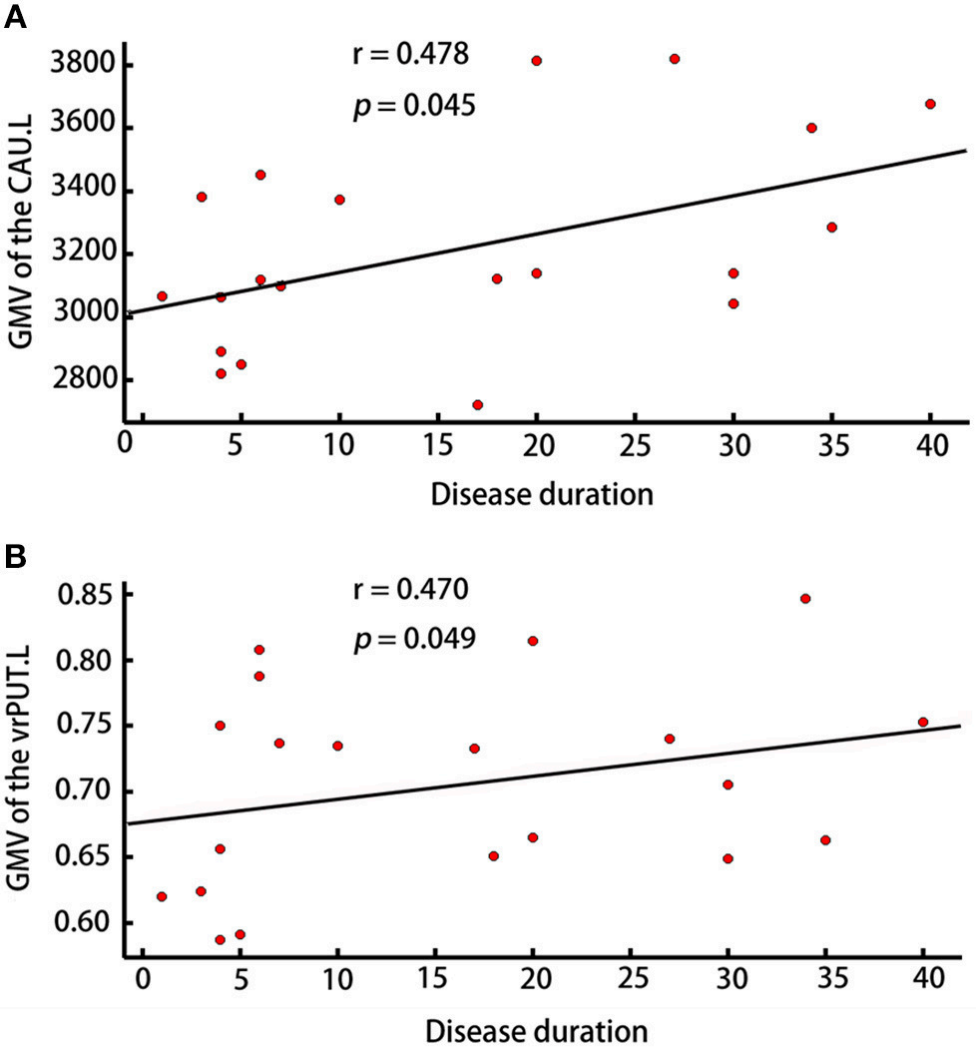
Correlation analyses. **(A)** Correlation analyses showed the GMV of CAU.L was correlated with disease duration in patients with RLS (*p* < 0.05). **(B)** Correlation analyses showed the GMV of vrPUT.L was correlated with disease duration in patients with RLS (*p* < 0.05).

The GMV of the vrPUT.L (*r* = 0.470, *p* = 0.049) and the CAU.L (*r* = 0.478, *p* = 0.045) were significantly correlated with disease duration of RLS (Figure 5).

## Discussion

In the present study, we found significant alterations of sub-cortical GMV, especially the PUT, rather than alterations of cortical GMV in patients with RLS compared to NC using both SBM and VBM. Further sub-regional analysis revealed that GMV alterations of PUT were mostly located in the dcPUT.L in patients with RLS. In addition, altered RSFC patterns of PUT were identified in patients with RLS compared to NC. Moreover, correlation analyses showed that the GMV of CAU.L and vrPUT.L were positively correlated with disease duration in patients with RLS.

Interestingly, combing the SBM and VBM analyses, the present study only found subcortical GMV alteration (i.e., PUT) in patients with RLS. Inconsistent with previous MRI studies, which not only found cortical GMV alteration in brain, like thalamus pulvinar (12), hippocampal gyrus (27) and ventral hippocampus (14), but also found cortical GMV alteration in primary somatosensory cortex, primary motor cortex, precentral gyrus etc. (13). Except the factors like medical treatment, methodological differences, the difference in the development process of RLS might provide major explanations for the inconsistence. Specifically, a series of iron-sensitive MRI studies found that the low iron levels in the dopaminergic related areas (like substantia nigra, striatum) might account the pathological process of RLS (28–30). At the meanwhile, positron emission tomography (PET) and photon emission computed tomography (SPECT) studies also found the dysfunction of dopaminergic pathways in the patients with RLS, in which nigrostriatal dopamine region (PUT) is involved (31). These findings might imply that: (1) the changes of neurotransmitter or the low level of iron components cause the abnormity of neural conduction; with the long course of RLS disease, these abnormities further result in the specific structural changes in these particular areas; (2) considering that only sub-cortical GMV changes were found in the present study, we hypothesized that the structural alterations of RLS patients are primarily changed in sub-cortical areas, especially in the PUT.

The PUT is a part of basal ganglia, which is interconnected to the substantia nigra, the globus pallidus, the claustrum and the thalamus, and works in conjunction with them to regulate motor behaviors (32–34) through various pathways. Astrakas et al. (10) and Margariti et al. (6) reported activation in the PUT when RLS symptoms were present. In addition, Connor et al. (35) also reported altered dopaminergic profile in the PUT in patients with RLS. Furthermore, a resting state fMRI study found the different RSFC patterns in the sub-divisions of PUT (23). In the direct comparisons, dcPUT showed the greater correlation with premotor cortices than drPUT; drPUT had the greater correlation with dorsal anterior cingulate gyrus (ACC) than dcPUT; while vrPUT showed the greater correlation with the dorsal lateral prefrontal cortex and rostral ACC than dcPUT and drPUT. In the present study, sub-regional GMV analysis revealed increased GMV in the dcPUT, which might suggest that dcPUT was most directly related to the dysfunction of the motor in the RLS. Therefore, these results suggested that this region was more vulnerable in the patients with RLS and might be used as potential biomarkers for the early diagnosis of RLS.

In addition to the structural changes in dcPUT.L, decreased RSFC between sub-divisions of PUT and other brain structures were identified in the RLS. These brain regions included ACC, precentral gyrus, frontal pole, superior frontal gyrus, CAU and cerebellum. ACC as a part of the brain's limbic system, it has been thought as a functional unit in constellation of other brain areas (36). The decreased RSFC between sub-regions of PUT and ACC might play a role in movement dysfunction in patients with RLS. In addition, several studies have revealed that regional volume reduction in ACC is related to depressive symptoms at sub-clinical level (37–39), which might also suggest that the altered RSFC was related to depression in patients with RLS. As precentral gyrus, superior frontal gyrus, and frontal pole are related to sensory and motor, it might imply that the altered RSFC in these areas were associated with the movement dysfunctions in patients with RLS. Previous studies have identified activation in the primary motor and somatosensory cortex in patients with RLS patients (6, 8, 10, 11). The CAU and the PUT consist of the dorsal striatum, which primarily mediates cognition involving motor function, certain executive functions (e.g., inhibitory control), and stimulus-response learning (40). The dysfunction between CAU and PUT might also provide some explanation to the symptom of RLS. Except for the decreased RSFC, we also found increased RSFC between drPUT.R and the right cerebellum. Margariti et al. (6) found activation in the bilateral cerebellum in patients with RLS. Furthermore, Chang et al. (15) found decreased GMV in the cerebellum. The cerebellum is important for sensory processing (41), and Chang et al. (15) thought the cerebellum might act as an integral network and inhibit the processing of uncomfortable sensory symptoms of RLS.

Although the brainstem was not included in the SBM analyses in the present study, VBM analyses revealed decreased GMV in the bilateral brainstem in the patients with RLS compared to NC. According to a previous diffusion tensor imaging study (42), the brainstem might play a conduction role by providing dopaminergic axons to the spinal cord and projecting into the cortex in the pathology of RLS. The alterations in the brainstem may cause the abnormity of the conduction which results in the unpleasant sensations in patients with RLS (bottom-up conduction) and the urge to move the legs (top-down conduction) (43, 44).

Additionally, the correlations between the GMV of CAU.L as well as the GMV of vrPUT.L and disease duration were identified in patients with RLS. As mentioned above, working with the PUT, the CAU primarily mediates cognition involving motor function, certain executive functions and stimulus-response learning (40). Therefore, it is reasonable to speculate that these two regions were associated with the pathology of RLS, particular with the movement dysfunction.

Two main limitations should be stressed in the present study. First, the sample size is modest; a larger sample size is needed to further reveal the pathological progress of RLS in the future. Second, as the sample size is limited, we cannot distinguish the different stages of the disease. The further division of duration might provide more information about the development process of RLS.

## Conclusions

In conclusion, we found alterations of sub-cortical GMV especially in bilateral PUT rather than cortical GMV in patients with RLS using both SBM and VBM. These results might imply that the primarily affected areas are located in sub-cortical areas especially in particular sub-regions of PUT by the pathologic process of RLS, which might be used as potential biomarkers for the early diagnosis of RLS.

## Author Contributions

CL and JX designed the study. HL, ZX, and YW participated in the data collection. TL and JX carried out the statistical analysis and wrote the paper. QH and BX assisted with writing the article. All authors read and approved the final manuscript.

### Conflict of interest statement

The authors declare that the research was conducted in the absence of any commercial or financial relationships that could be construed as a potential conflict of interest. The reviewer MS and the handling editor declared their shared affiliation.
